# Investigation of PACAP Fragments and Related Peptides in Chronic Retinal Hypoperfusion

**DOI:** 10.1155/2014/563812

**Published:** 2014-05-12

**Authors:** Dora Werling, Dora Reglodi, Peter Kiss, Gabor Toth, Krisztina Szabadfi, Andrea Tamas, Zsolt Biro, Tamas Atlasz

**Affiliations:** ^1^Department of Anatomy, PTE-MTA “Lendulet” PACAP Research Team, University of Pecs, Szigeti ut 12, Pecs 7624, Hungary; ^2^Department of Medical Chemistry, University of Szeged, Dom Ter 8, Szeged 6720, Hungary; ^3^Department of Experimental Zoology and Neurobiology, University of Pecs, Ifjusag Utja 6, Pecs 7624, Hungary; ^4^Department of Ophthalmology, University of Pecs, Nyar Utca 8, Pecs 7624, Hungary; ^5^Department of Sportbiology, University of Pecs, Ifjusag Utja 6, Pecs 7624, Hungary; ^6^Janos Szentagothai Research Center, University of Pecs, Ifjusag Utja 20, Pecs 7624, Hungary

## Abstract

Pituitary adenylate cyclase activating polypeptide (PACAP) has neuroprotective effects in different neuronal and retinal injuries. Retinal ischemia can be effectively modelled by permanent bilateral common carotid artery occlusion (BCCAO), which causes chronic hypoperfusion-induced degeneration in the entire rat retina. The retinoprotective effect of PACAP 1-38 and VIP is well-established in ischemic retinopathy. However, little is known about the effects of related peptides and PACAP fragments in ischemic retinopathy. The aim of the present study was to investigate the potential retinoprotective effects of different PACAP fragments (PACAP 4-13, 4-22, 6-10, 6-15, 11-15, and 20-31) and related peptides (secretin, glucagon) in BCCAO-induced ischemic retinopathy. Wistar rats (3-4 months old) were used in the experiment. After performing BCCAO, the right eyes of the animals were treated with PACAP fragments or related peptides intravitreal (100 pM), while the left eyes were injected with saline serving as control eyes. Sham-operated (without BCCAO) rats received the same treatment. Routine histology was performed 2 weeks after the surgery; cells were counted and the thickness of retinal layers was compared. Our results revealed significant neuroprotection by PACAP 1-38 but did not reveal retinoprotective effect of the PACAP fragments or related peptides. These results suggest that PACAP 1-38 has the greatest efficacy in ischemic retinopathy.

## 1. Introduction

Pituitary adenylate cyclase activating polypeptide (PACAP) is a neuropeptide with widespread occurrence in various organs and diverse effects both in the nervous system and in the periphery [1, 2]. PACAP is strongly expressed in the central nervous system, where it exerts several effects such that it is a central regulator of circadian rhythmic activities [3], plays a role in memory formation [4] and psychiatric processes [5], and is involved in central feeding control [6].

PACAP is also known to be expressed in the retina, along with its receptors (PAC1, VPAC1, and VPAC2 receptors). Numerous studies have provided evidence that the neuroprotective effects are mainly mediated by the PAC1 receptor and diverging downstream pathways upon its activation [7–9]. The PAC1 receptor has several splice variants, which can mediate not only different but also opposing effects [10]. Several research groups have proven that PACAP has strong protective effects against various retinal injuries. In vitro, it protects retinal explants against excitotoxic injury [11] and retinal pigment epithelial cells against oxidative stress [12]. In vivo retina studies show that PACAP protects against NMDA- and MSG-induced excitotoxic damage [13, 14], UV light-induced lesion [15], and optic nerve lesion [16]. Recent studies show that PACAP is also protective in diabetic retinopathy [17]. Retinal ischemia can be induced by several methods, mimicking pathological features seen in human glaucoma-related retinal lesions, in chronic retinal hypoperfusion, and in other types of retinal lesions accompanied by ischemia. The protective role of PACAP has also been proven in models of retinal ischemia [18, 19]. Based on these studies, evidence for the protective effects of PACAP in the retina is now well established [20].

The bioavailability and fast degradation of PACAP limit its therapeutic use and attention has been drawn to the application of shorter fragments and/or analogs. N-terminally shorter fragments of PACAP usually have antagonistic effects, but some reports have documented agonistic behavior, depending on the cell/tissue type [7–9, 21, 22]. In contrast, C-terminally shorter fragments usually differ in the strength of receptorial binding [7–9]. Therefore, it is necessary to test whether shorter PACAP fragments have any effect, ameliorating or damaging, on retinal lesions. We have previously shown that PACAP 6-38, the most widely used antagonist of PACAP, has an aggravating effect on retinal excitotoxic lesion [23]. However, it is not known, whether the shorter fragments of PACAP have any effect on the retina. Therefore, the first aim of our present study was to examine the effects of PACAP fragments 4-13, 4-22, 6-10, 6-15, 11-15, and 20-31 on chronic retinal hypoperfusion induced by bilateral carotid artery occlusion. Possible fragments to be tested were selected in order to cover a wide range of the molecule, from the N-terminal to the C-terminal and middle region of the peptide. In addition, the N-terminal fragments show a high similarity with the structure of VIP. Furthermore, the 4-13 domain, for example, shows high selectivity to the PAC1 receptor, which is mainly responsible for the neuroprotective effects of PACAP [7–9]. In a recent study we have shown that the peptide most closely related to PACAP, namely, vasoactive intestinal peptide (VIP), is also protective in retinal ischemia. However, to achieve a degree of neuroprotection similar to PACAP, higher doses are required [24]. Other members of the peptide family have not been tested in retinal ischemia so far. Therefore, the second aim of the present study was to investigate whether secretin and glucagon have any effect on a rat model of retinal ischemia.

## 2. Materials and Methods

Experimental animals were derived from a local colony of Wistar rats. Animals were housed in individual cages, fed, and watered ad libitum, under light/dark cycles of 12/12 h. All animal procedures complied with the University of Pecs (number BA02/2000-15024/2011) for the ethical use of animals. Adult male rats (*n* = 32) weighing 250–300 g were exposed to permanent bilateral common carotid artery occlusion (BCCAO) under isoflurane anesthesia and both common carotid arteries were ligated with a 3-0 filament through a midline incision [19]. A group of animals underwent anesthesia and all steps of the surgical procedure, except ligation of the carotid arteries. These animals served as sham-operated animals (*n* = 7). Immediately following the operation, PACAP 1-38 or its fragments (PACAP 4-13, 4-22, 6-10, 6-15, 11-15, and 20-31) or other members of the peptide family such as secretin and glucagon (100 pmol/5 µL) were injected intravitreally using 30-gauge Hamilton syringe into the right vitreous body of animals. The left eyes received the same volumes of vehicle treatment (physiological saline) and served as ischemic eyes. Rats were sacrificed with an overdose of anesthetic after 2 weeks of BCCAO and the eyes were processed for histological analysis. Briefly, the eyes were dissected in PBS and fixed in 4% paraformaldehyde dissolved in 0.1 M PB. Following fixation, tissues were embedded in Durcupan ACM resin, cut at 2 *μ*m, and stained with toluidine blue. Four tissue blocks from at least four animals were prepared and central retinal areas within 1 mm from the optic nerve were used for measurements (*n* = 5 measurements from one tissue block). Photographs were taken with a digital CCD camera using the Spot program. Sections where the GCL appeared thicker than a single cell row were omitted from evaluation. The following parameters were measured: (i) the width of the outer and inner nuclear and plexiform layers (ONL, OPL, INL, and IPL, resp.); (ii) the number of cells/100 µm section length in the ganglion cell layer (GCL); (iii) the number of cells/500 µm^2^ area in the INL. Results are presented as mean ± SEM. Statistical comparisons were made using the ANOVA test followed by Tukey-B's* post hoc* analysis.

## 3. Results and Discussion

**Figure 1:**
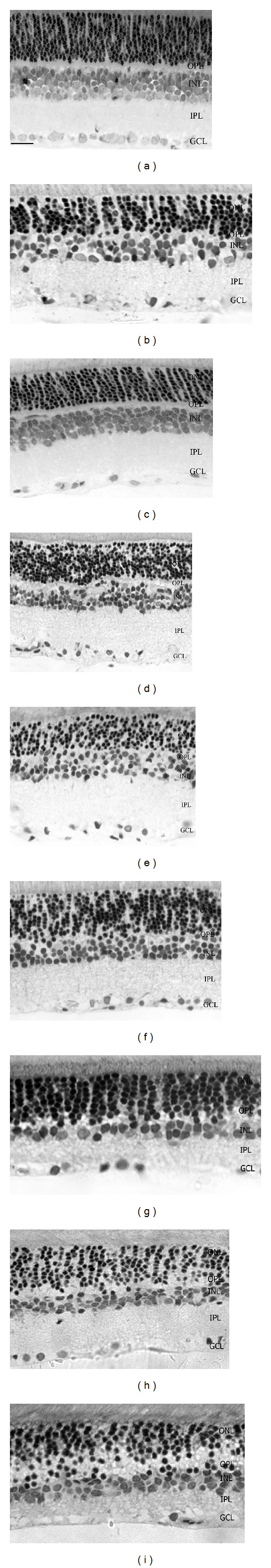
Representative light microphotographs of retinal sections. Retinal tissue from SHAM-operated animal (a) compared to BCCAO retinas (b). Representative sections from retinas treated with PACAP 1-38 (c) and PACAP fragments: PACAP 4-22 (d), 6-15 (e), 11-15 (f), 20-31 (g), 6-10 (h), and 4-13 (i). In contrast to PACAP 1-38 (c), the PACAP fragments (d–f) did not ameliorate the BCCAO-induced retinal degeneration. On the contrary, treatment with some fragments led to a more pronounced degeneration, which was shown by the width of the retinal layers and the neural profiles in the ONL and INL. ONL: outer nuclear layer; OPL: outer plexiform layer; INL: inner nuclear layer; IPL: inner plexiform layer; GCL: ganglion cell layer (scale bar: 20 µm).

**Figure 2:**
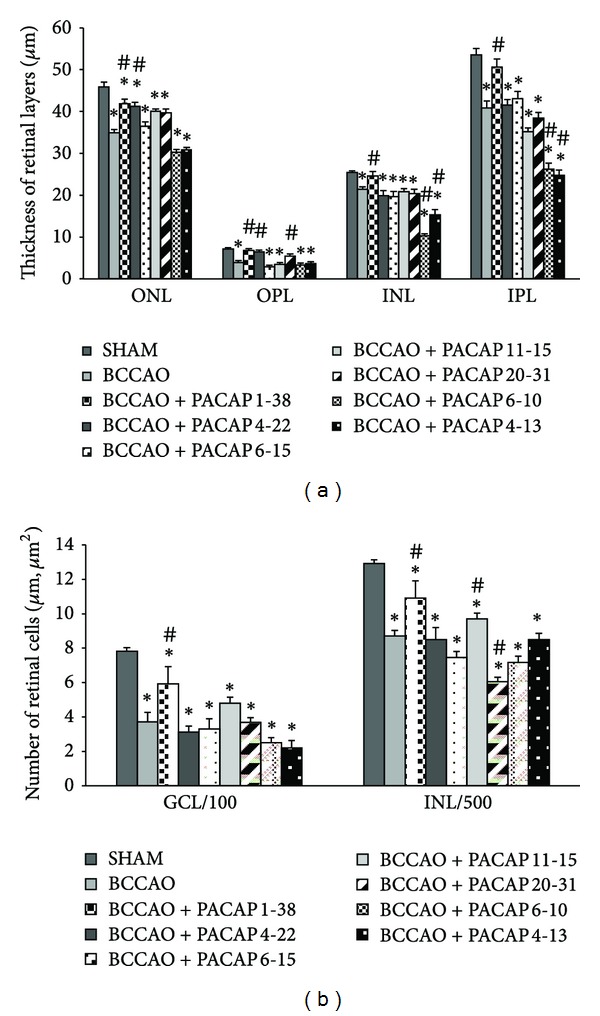
Morphometric analysis of retinal layers (a) and number of cells/100 µm GCL length and number of cells/500 µm^2^ INL (b) in SHAM operated animals, with BCCAO and with BCCAO + different PACAP fragments. We did not detect retinoprotective effects after the injection of the PACAP fragments (PACAP 4-22, 6-15, 11-15, 20-31, 6-10, and 4-13). ONL: outer nuclear layer; OPL: outer plexiform layer; INL: inner nuclear layer; IPL: inner plexiform layer; GCL: ganglion cell layer. *P < 0.05 compared to SHAM retinas; ^#^ *P* < 0.05 compared to BCCAO retinas.

BCCAO resulted in severely reduced thickness of retinal layers as observed two weeks after ligation compared to sham-operated controls (Figure 1(a)). Marks of degeneration with individual variations are visible in all retinal layers (Figure 1(b)). Morphometric analysis revealed that the most pronounced reduction in thickness in retinas with BCCAO was found in the OPL (Figure 2). The photoreceptor layer was also reduced in thickness (Figure 1(b)). This layer was significantly thinner than that of the control specimens (Figure 2). Many cells in the ganglion cell layer (GCL) also suffered degeneration, shown by necrotic cells in this layer (Figures 1 and 2). This fact is well reflected in the reduced number of cells in the GCL (Figure 2). Intraocular PACAP 1-38 treatment following BCCAO led to nearly healthy appearance of the retinal layers (Figure 1(c)). This is also well supported by the morphometric measurements. The thickness of the major retinal layers was almost identical with that of the sham-operated animals and was significantly larger than that of control ischemic ones (Figure 2). This was especially conspicuous in the OPL, which disappeared, and the ONL and INL layers were fused in several control animals and were preserved in all PACAP 1-38 injected animals. However, the number of cells in the GCL seemed to be lower than in the sham-operated animals (Figure 2). Indeed, some degenerating cells could be discerned in the GCL in these preparations. This may have led to a decrease in cell numbers.

**Figure 3:**
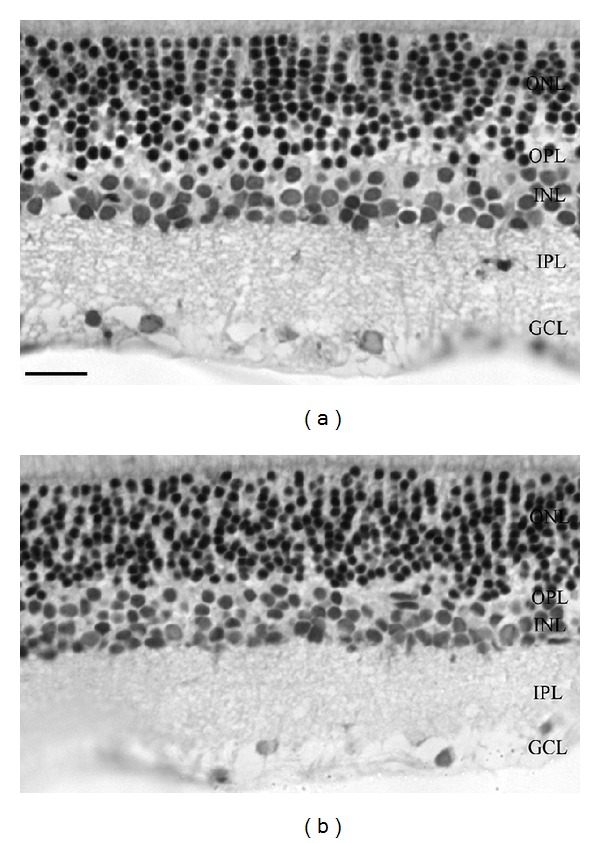
Light microphotographs of retinal sections (toluidine blue). Representative retinas from BCCAO + secretin (a) and BCCAO + glucagon (b). The whole retina structure (especially ONL and INL) was damaged by BCCAO compared to SHAM retinas. The PACAP related peptide secretin (a) and glucagon (b) did not ameliorate the ischemic degeneration (scale bar: 20 µm).

**Figure 4:**
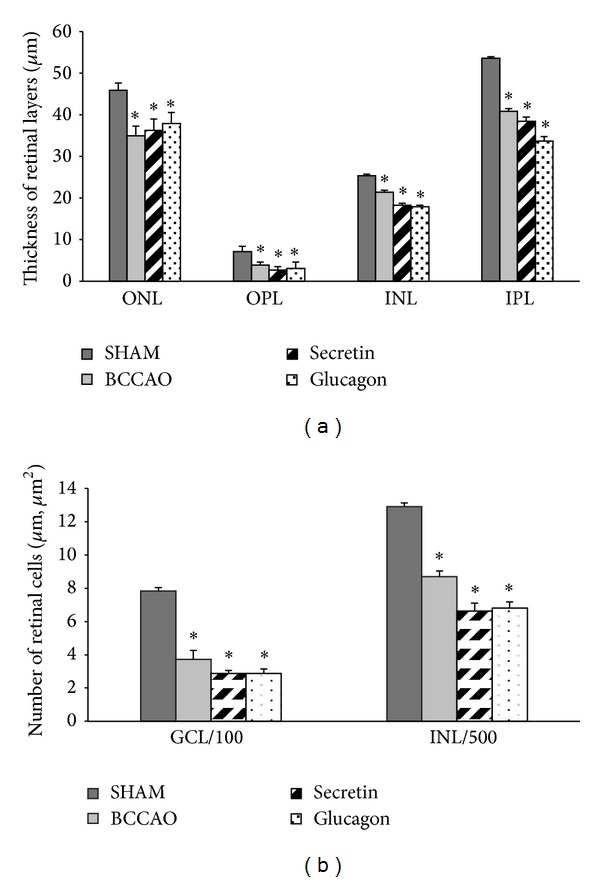
Morphometric analysis of retinal layers (a) and number of cells/100 µm GCL length and number of cells/500 µm^2^ INL (b) in secretin- and glucagon-treated retinas. The retinoprotective effects of secretin or glucagon were not quantified by the thickness (*μ*m) of retinal layers (a) OPL, INL, and IPL and number of cells/100 *μ*m GCL length or the number of cells/500 *μ*m^2^ in the INL (b). ONL: outer nuclear layer; OPL: outer plexiform layer; INL: inner nuclear layer; IPL: inner plexiform layer. *P < 0.05 compared to SHAM retinas.

Intravitreal injection of different PACAP fragments (PACAP 4-22, 6-15, 11-15, 20-31, 6-10, and 4-13) did not ameliorate the ischemic damage of the retina (Figures 1(d)–1(i)). In all samples, nuclear layers (ONL, INL) and plexiform layers (OPL, IPL) were reduced, and the cell number in the GCL was significantly less than in sham-operated controls. However, injection with PACAP 6-10 and 4-13 resulted in further reduction of the inner plexiform and nuclear layers (Figures 1(h) and 1(i)). Treatment with members of the PACAP-related peptide superfamily, glucagon and secretin, led to no aggravation or amelioration of the ischemic retinal lesion (Figure 3). These observations were confirmed by morphometric measurements (Figure 4).

PACAP is a member of the secretin/glucagon/VIP superfamily of peptides. PACAP and its receptors occur in the retina [25]. PACAP is thought to play an important role in visual processing, as it is an important cotransmitter in the retinohypothalamic tract that is the major input for the biological clock situated in the suprachiasmatic nucleus [26]. Besides its supposed physiological actions in the retina, PACAP is a well-established retinoprotective peptide. Endogenous protection is proven by the increased vulnerability of the retina in mice lacking endogenous PACAP in ischemic retinal lesion [2] and in NMDA-induced excitotoxic injury [14]. These observations are further supported by the increased apoptotic activity in retinas injected by the PACAP antagonist PACAP 6-38 [27]. As PACAP is a well-accepted retinoprotective agent in models of different retinal injuries, several studies have attempted to explore the molecular mechanisms explaining this strong retinoprotection [27]. We have shown that PACAP activates the antiapoptotic, while it inhibits the proapoptotic signaling pathways in retinal toxic lesions [27]. In retinal ischemia, we have shown that PACAP administration provides protection by involving Akt, MAPK pathways, and anti-inflammatory actions [28].

Given the very potent actions of PACAP in retinal and other pathologies, there is an urging demand for developing novel analogues and/or fragments to increase the bioavailability and stability of the peptide for future potential therapeutic use [7, 8]. Binding PACAP to other carrier molecules is a possibility to increase its capacity to traverse through membranes and to exert cytoprotective effects [29]. It has also been shown that PACAP is able to pass through cell membranes and convey nonreceptor mediated effects [30]. This has raised the possibility of using PACAP fragments also for supporting transport of other nonpenetrable molecules [31]. Several analogues have also been developed for these purposes [7, 8, 32]. Interestingly, however, a stable analogue did not provide stronger neuroprotection in a stroke model than the natural peptide [33]. Unfortunately, all these efforts do not seem to lead to obtaining of a more effective molecule than the original peptide or to obtaining of a biologically more stable analogue that has the same protective property as PACAP 1-38. The evolutionary pressure on the form of PACAP 1-38 seems to be very strong, as effects found in vertebrates have also been described in invertebrates [7]. Although a lot of pharmacological and receptor binding studies have been performed with fragments/analogues of PACAP, only few studies have tested the biological efficacy of these forms [33]. Since some studies suggest that there are unconventional binding sites, unusual behavior of the peptide forms in some tissues and even implies the existence of unknown receptor splice variants [21, 22, 34]; it is important to test the biological effects of fragments in a system that is well standardized in numerous earlier studies. Our present results show that the shorter fragments of PACAP, as expected, do not provide any neuroprotection. In addition, we can confirm that most of them do not deteriorate the degree of damage either, although some parameters were worsened in cases of PACAP 6-10 and 4-13. This is in accordance with our earlier observations showing that PACAP antagonists PACAP 6-38 and 6-27 lead to aggravated retinal lesion in an MSG-induced retinal injury model [23].

Relatively little is known about other members of the peptide family in the retina. Some other members of this family have been localized in the vertebrate retina and several functions have been attributed to them [35, 36]. Glucagonergic amacrine cells represent a small subpopulation of the amacrine cells possibly playing a role in the visual processing [37, 38]. Other studies have shown that glucagon plays a role in eye bulb growth and glucagon is suggested to be an endogenous mediator of emmetropization [39]. An early study has shown that glucagon increases cAMP in chick retinal Muller cells, similar to the actions of VIP [40]. The protective effects of secretin and glucagon in cerebral pathologies have been implicated in a few studies [41–43]. In cerebral ischemia, glucagon has been shown to exert neuroprotective effects in vivo [41]. It has not been investigated so far whether secretin or glucagon is protective in retinal lesions. Based on our present study, neither glucagon nor secretin has protective action in retinal ischemia. Based on our present and previous results, a strong neuroprotective effect is a specific PACAP-action, since VIP could exert its protective effects only in much higher doses, while secretin and glucagon did not have any effects.

Our present results confirm that the natural form of the peptide, PACAP 1-38, is the most effective peptide form in retinal ischemia, and the 38 amino acid form of the peptide cannot be replaced by another fragment or another member of the peptide family that we know of. Our results further support the potent retinoprotective effects of PACAP and call for further studies to establish the future possible clinical introduction of PACAP-related retinoprotective therapeutic approach.
